# Tumeur brune de la mandibule révélatrice d'une hyperparathyroïdie primaire: à propos d'un cas

**DOI:** 10.11604/pamj.2014.18.200.4844

**Published:** 2014-07-05

**Authors:** Abdelfattah Aljalil, Brahim Bouaity

**Affiliations:** 1 Service d'ORL et de CCF, Hôpital Militaire Avicenne, Marrakech, Maroc

**Keywords:** Tumeur brune, mandibule, hyperparathyroïdie primaire, Brown tumor, mandible, primary hyperparathyroidism

## Abstract

Les tumeurs brunes sont des manifestations osseuses classiques des hyperparathyroïdies (HPT). Elles surviennent habituellement lors des formes sévères accompagnées de signes de résorption osseuse périostée. La mandibule en constitue une localisation habituelle mais rarement révélatrice. Leur traitement repose essentiellement sur la paratharoïdectomie. Nous décrivons un cas de tumeur brune de la mandibule chez un patient de 46 ans; ainsi que son évolution favorable après para thyroïdectomie.

## Introduction

Les tumeurs brunes font partie des manifestations osseuses classiques des hyperparathyroïdies (HPT) primitives ou secondaires sévères [1, 2, 3]. Elles intéressent préférentiellement les os de la face, notamment la mandibule. Elles peuvent également atteindre le bassin, les côtes et les fémurs. Nous décrivons une localisation mandibulaire révélant une hyperparathyroïdie primaire chez un patient de 46ans ainsi que son évolution après parathyroïdectomie.

## Patient et observation

**Figure 1:**
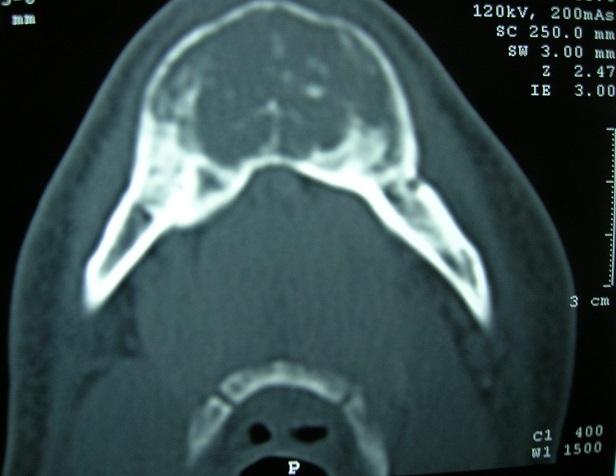
Mandibulaire en coupe axiale montrant le processus tumoral de la mandibule avec des trabéculations intra lésionnelles et une corticale soufflée par endroit

**Figure 2:**
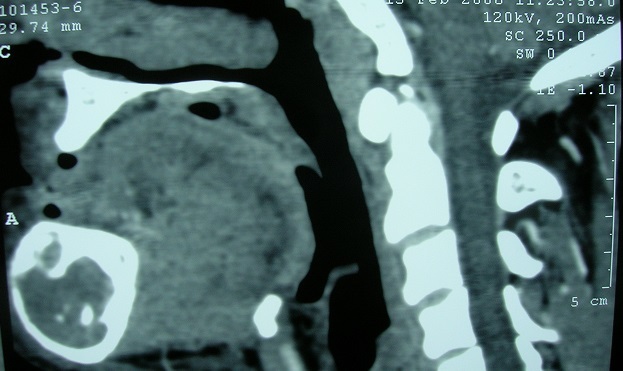
TDM mandibulaire en reconstruction sagittale montrant le même processus tumoral au dépends de la mandibule

**Figure 3:**
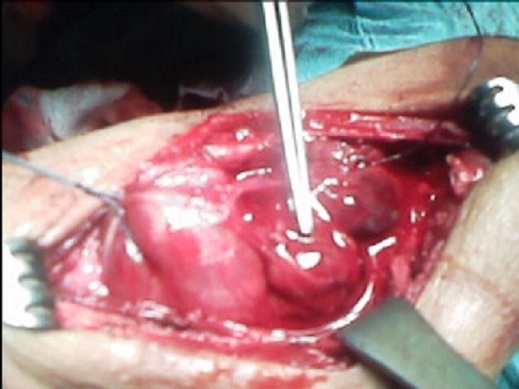
Vue opératoire montrant l'adénome parathyroïdien inférieur gauche

Monsieur BH, âgé de 46 ans, ayant une notion de lithiase urinaire dans les antécédents, traitée par hydrothérapie et qui présentait depuis 2 ans une tuméfaction mandibulaire augmentant progressivement de volume sans signes accompagnateurs. A l'occasion d'une visite médicale systématique, une hypertension artérielle a été découverte associée à une insuffisance rénale pour laquelle le patient a été adressé en néphrologie. L'examen d'entrée mettait en évidence cette tuméfaction mandibulaire de 3cm/3cm, de consistance ferme, indolore; et sans signes inflammatoires en regard. Un bilan biologique a été réalisé qui montrait une hypercalcémie à125mg/l (N:88-102), une hypophosphorémie à 23 mg/l (N:27-45) avec altération de la fonction rénale: urée à 1.34g/l (N:0.1-0.5) et créatinine à 22 (N:7-12). La tomodensitométrie (TDM) mandibulaire montrait la présence d'un processus tumoral cloisonné ostéolytique du corps mandibulaire mesurant 3.5/5cm (Figure 1, Figure 2). Une échographie cervicale objectivait un gros adénome parathyroïdien solitaire (inférieur gauche) et le dosage plasmatique de la parathormone 1-84 était élevé à 1091 pg/ml (N <72). L'exérèse de l'adénome a été réalisée facilement à travers un abord cervical unilatéral avec une exploration ciblée et minimaliste (Figure 3). Les suites opératoires ont été simples avec un retour à la normale du taux de la PTH 1-84 et du bilan phosphocalcique.

## Discussion

La fréquence des manifestations osseuses au cours de l′HPT primaire est de 10 à 15%. La tumeur brune représente un aspect rare de ses manifestations (2 à 3%), et elle est exceptionnellement révé1atrice [4]. Ses localisations sont le bassin, les cotes, les diaphyses des os longs, les métacarpiens et phalanges, la mandibule [1–3, 5, 6]; plus rarement le rachis et la base du crâne. Des formes pluri focales sont possibles [1–3, 5], ce qui doit les faire rechercher systématiquement sur les sites à risque (diaphyses des membres inférieurs, rachis).

Les signes radiologiques (TDM +++) d'hyperparathyroïdie les plus fréquents sont la résorption osseuse sous périostée (houppes et diaphyses phalangiennes), sous chondrale (acromioclaviculaires) et l ‘ostéoporose granuleuse du crâne. La présentation radiologique des tumeurs brunes peut être trompeuse: lésions ostéolytiques ayant parfois des contours polycycliques, corticale amincie, effacée ou soufflée [7]. Ces signes peuvent évoquer une ostéolyse tumorale maligne, une tumeur à cellules géantes multicentriques [3, 7, 8], voire une dysplasie fibreuse [3, 8]. L'aspect scintigraphique osseux est une hyperfixation voire une hypofixation en cas de processus lytique pur [8]. Quelque soit la localisation, et en cas de doute diagnostique, la biopsie de la lésion peut être nécessaire. Cependant le contexte clinique et l'existence d'une HPT biologique franche suffisent en général [4].

Nous avons utilisé un traitement par biphosphonate en préopératoire pour limiter l'extension de l'ostéolyse tumorale pouvant survenir à l'occasion d'une élévation transitoire de la parathormone lors de la parathyroidectomie. Le traitement médical à base de vitamine D, n'a été rapporté que dans certaines HPT secondaires sévères avec tumeur brune chez les hémodialysés - qui nécessite d'abord le maintien d'une phosphorémie idéalement inférieure à 2 mmol/L- [4], mais l'efficacité à long terme n'est pas acquise. Les causes d’échec sont le mauvais contrôle de la phosphorémie, une hypercalcémie ne permettant pas d'utiliser les fortes doses de vitamine D nécessaires [4, 9], ainsi que l'existence d'un adénome parathyroïdien. Silverman et coll [10] ont reporté qu'il n'est pas nécessaire de faire l'excision d'une tumeur brune une fois l'HPT est résolue, toutefois Steinbach et coll [11] ont rapporté que ces tumeurs peuvent être traitées par radiothérapie ou curetage.

Cependant, beaucoup d'auteurs préconisent que les tumeurs brunes devraient être généralement traitées par une parfaite paratharoïdectomie première qui facilite leur régression et l'excision ne se conçoit qu'en cas de résidu tumoral [11]. Dans notre cas, il y avait une régression tumorale parfaite après parathyroidectomie sans aucun résidu. Nous soulignons enfin l'intérêt d'un traitement spécifique adapté dans lequel la place de la parathyroidectomie est établie et des biphosphonates reste à définir.

## Conclusion

Les tumeurs brunes de la mandibule sont des manifestations osseuses rarement révélatrices des HPT primaires. Leur découverte impose l'exploration des glandes parathyroïdes sièges le plus souvent d'adénome. La parathyroïdectomie reste le traitement de référence de cette affection.
